# Purified Human Synovium Mesenchymal Stem Cells as a Good Resource for Cartilage Regeneration

**DOI:** 10.1371/journal.pone.0129096

**Published:** 2015-06-08

**Authors:** Yusuke Ogata, Yo Mabuchi, Mayu Yoshida, Eriko Grace Suto, Nobuharu Suzuki, Takeshi Muneta, Ichiro Sekiya, Chihiro Akazawa

**Affiliations:** 1 Department of Biochemistry and Biophysics, Graduate School of Health Care Sciences, Tokyo Medical and Dental University, Tokyo, Japan; 2 Department of Joint Surgery and Sport Medicine, Graduate School of Medicine, Tokyo Medical and Dental University, Tokyo, Japan; 3 Center for Stem Cell and Regenerative Medicine, Tokyo Medical and Dental University, Tokyo, Japan; RWTH Aachen University Medical School, GERMANY

## Abstract

Mesenchymal stem cells (MSCs) have the ability to differentiate into a variety of lineages and to renew themselves without malignant changes, and thus hold potential for many clinical applications. However, it has not been well characterized how different the properties of MSCs are depending on the tissue source in which they resided. We previously reported a novel technique for the prospective MSC isolation from bone marrow, and revealed that a combination of cell surface markers (LNGFR and THY-1) allows the isolation of highly enriched MSC populations. In this study, we isolated LNGFR^+^ THY-1 ^+^ MSCs from synovium using flow cytometry. The results show that the synovium tissue contained a significantly larger percentage of LNGFR ^+^ THY-1 ^+^ MSCs. We examined the colony formation and differentiation abilities of bone marrow-derived MSCs (BM-MSCs) and synovium-derived MSCs (SYN-MSCs) isolated from the same patients. Both types of MSCs exhibited a marked propensity to differentiate into specific lineages. BM-MSCs were preferentially differentiated into bone, while in the SYN-MSC culture, enhanced adipogenic and chondrogenic differentiation was observed. These data suggest that the tissue from which MSCs are isolated should be tailored according to their intended clinical therapeutic application.

## Introduction

Mesenchymal stem cells (MSCs) are self-renewing cells that can differentiate into osteoblasts, chondrocytes, and adipocytes [1–4]. These cells are found in various human tissues, including bone marrow (BM), synovium (SYN), placenta, and adipose tissue [3,5–9]. The characterization of MSCs isolated from those various tissues remains relatively unexplored. Because MSCs display no tumorigenicity, therefore they are currently used in clinical applications [10,11]. Number of clinical studies have been performed using MSCs to cure a variety of diseases [12–15]. Because origin tissues and isolation techniques are not unified, these clinical trials led to variable results. It has not been well-characterized how the differentiation potential of MSCs differs according to the tissue from which they are derived.

There have been several reports describing that synovium-derived MSCs (SYN-MSCs) showed a higher colony-forming efficiency than MSCs derived from bone marrow (BM-MSCs) [16,17]. Because SYN-MSCs display a great potential to differentiate into chondrocytes, SYN-MSCs are one of the best candidates for the repair of damaged cartilage [18,19]. A few reports have characterized the surface markers of SYN-MSCs. Cultured SYN-MSCs express such as CD44, CD90 (known as THY-1), CD105, and CD166, which are also found on fibroblasts and stromal lineages, and do not express hematopoietic and endothelial specific markers including CD45, CD253a, and CD31 [16,17]. We recently reported combination of novel markers for prospective MSC isolation and revealed that a combination of cell surface markers (low-affinity nerve growth factor receptor (LNGFR) and THY-1) allows the isolation of highly enriched MSC populations [20]. We also showed that LNGFR^+^THY-1^+^ cells, containing MSC-like cells, are present in placenta and adipose tissue [20].

In the current study, we freshly isolated MSCs from synovium and bone using surface markers, LNGFR and THY-1. We show that the MSCs existed high frequency in the synovium tissue, and the pattern of surface marker expression was similar between SYN- and BM- MSCs. BM-MSCs have a preference to differentiate to bone, while SYN-MSCs retains preference to both adipogenic and chondrogenic differentiation. Our results suggest that the tissue from source of MSCs should be tailored according to their intended therapeutic application.

## Materials and Methods

### Ethics information

Synovium and bone fragments were harvested from donors during total knee arthroplasty surgery at Tokyo Medical and Dental University Hospital. In total, 10 biological samples were used for the experiments. All experiments were approved by the local Institutional Review Board of Tokyo Medical and Dental University (No. 1030) and all study participants provided written informed consent. Tissue sample information and actual value of isolated LNGFR^+^THY-1^+^ cells for experiments show in S1 Table.

### Tissue preparation

Synovium was digested with 2 mg/mL collagenase (Wako), 3 mg/mL dispase (Wako), and 25 μg/mL deoxyribonuclease I (Sigma-Aldrich) prepared in Dulbecco’s Modified Eagle’s Medium (DMEM, Life Technologies) with shaking at 37°C for 1 hour. Bone fragments were crushed with a pestle, after which the crushed bones were washed gently once in phosphate buffered saline (PBS) (for remove the marrow cells). Bone and bone fragments were incubated for 1 hour at 37°C in DMEM in the presence of 2 mg/mL collagenase (Wako Chemicals) and 25 μg/mL deoxyribonuclease I (Sigma-Aldrich). The cell suspensions (synovium and bone fragments) were filtered through a cell strainer (Falcon, 70 μm) to remove debris. After lyse the red blood cells, these cells were re-suspended in calcium- and magnesium-free Hank's Balanced Salt Solution (HBSS) (Gibco) supplemented with 2% FBS, 10 mM HEPES and 1% penicillin/streptomycin.

### Flow cytometric analysis and cell sorting

Digested cells were suspended in HBSS at a density of 1–5 × 10^7^ cells/mL and stained for 30 minutes on ice with THY-1-APC (BD Pharmingen) and LNGFR-PE (Miltenyi Biotec) antibodies for sorting. Cultured cells (at three and five passages) were harvested using cell-dissociation buffer (Gibco). Cells (1.0 × 10^5^) were suspended in ice-cold HBSS and stained for 30 minutes on ice with the monoclonal antibodies CD45-PE-Cy7 (Tonbo Biosciences), CD29-PE, CD31-PE-Cy7, CD44-PE, CD105-PE and CD166-PE (BioLegend) for cell surface analysis. Flow cytometric analysis and cell sorting were performed on a triple-laser Moflo system (Beckman Coulter) and data were analyzed using Flowjo software (Tree Star).

### Colony-forming unit-fibroblast (CFU-F) assay

The CFU-F assay was performed by culturing sorted 2,000 cells in a 100-mm dish for 14 days in culture medium, which consisted of DMEM supplemented with 20% fetal bovine serum (Hyclone), 1% penicillin/streptomycin (Gibco), and 5 ng/mL basic fibroblast growth factor (Repro Cell). The medium was changed every 3–4 days. Colonies containing more than 50 cells were counted.

### Cell differentiation

Cultured MSCs at three passages were harvested using Trypsin-EDTA (Gibco). For adipogenic differentiation, 1.0 × 10^4^ cells were transferred to a 24-well plate and cultured overnight in culture medium. Adherent cells were cultured in adipogenic induction medium (Lonza), which was changed every 3–4 days. After 14 days, oil red O staining (Muto Pure Chemicals) confirmed the differentiation of these cells into adipocytes. For osteogenic differentiation, 7.0 × 10^3^ cells were transferred to a 24-well plate and cultured overnight in culture medium. Adherent cells were cultured in osteogenic induction medium (Lonza), which was changed every 3–4 days. After 14 days, the differentiation of these cells into osteoblasts was assessed by alizarin red staining (Millipore). For chondrogenic differentiation, 5.0 × 10^5^ cells were transferred to a 15-mL tube and cultured in chondrogenic induction medium (Lonza) containing 10 ng/mL transforming growth factor-β3 (Lonza) and 500 ng/mL bone morphogenetic protein 6 (R&D Systems), which was changed every 3–4 days. After 21 days, chondrogenic differentiated cells were was analyzed by Toluidine blue (Wako) and Safranin O staining (TCI) staining.

### Real-time RT-PCR

After cell differentiation, total RNA was prepared with TRI reagent (Sigma-Aldrich) as described previously [21,22]. Complementary DNA was amplified using the StepOne Real-Time PCR System (Life Technologies) and normalized against β-actin expression (each gene were determined between 20–35 cycles). The experiments were performed averaged duplicates value as a data and using more than three independent biological samples. The probes used to identify gene expression of the key markers were confirmed to be human-specific sequence (TaqMan gene Expression Assays). All analyzed genes are listed in S2 Table. The PCR data using FAST SYBR Green showed in S1 Fig and S3 Table.

### Statistical analysis

Quantitative data are presented as means ± standard deviation of at least three independent experiments. For statistical analysis, data were evaluated using Paired T-test. In all cases, p-values of < 0.05 were considered significant.

## Results

### The LNGFR^+^THY-1^+^ population of synovium-derived cells is enriched in clonogenic cells

**Fig 1:**
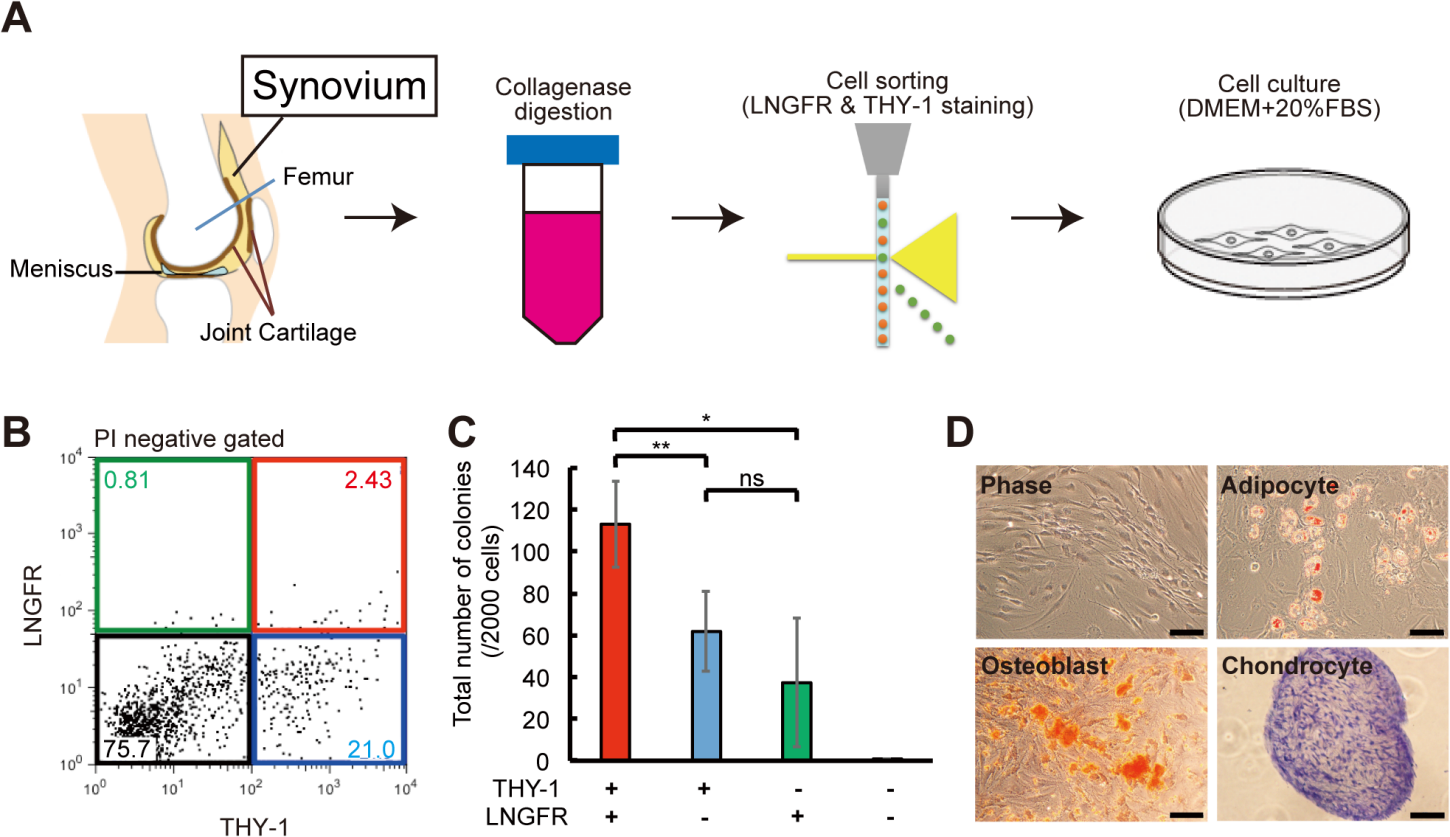
Isolation of LNGFR^+^THY-1^+^ MSCs from SYN. (A) Synovium (SYN) was harvested from patients undergoing total knee arthroplasty surgery and digested with collagenase and dispase. Mesenchymal stem cells (MSCs) were isolated using flow cytometry. DMEM, Dulbecco’s Modified Eagle’s Medium; FBS, fetal bovine serum. (B) Representative flow cytometric profile of SYN-derived cells stained for LNGFR and THY-1. Each population shows in LNGFR^+^/THY-1^+^(red), LNGFR^+^/THY^-^ (green), LNGFR^-^/THY^+^ (blue) and LNGFR^-^/THY^-^ (black) gates. PI, propidium iodide. (C) Number of colonies formed after 2 weeks of culturing 2,000 of the following cells: LNGFR^+^/THY-1^+^, LNGFR^+^/THY^-^, LNGFR^-^/THY^+^, and LNGFR^-^/THY^-^ (mean ± standard deviation, n = 4 per group; *p<0.05; **p < 0.01; ns, not significant). (D) Phase contrast micrographs of LNGFR^+^THY-1^+^ cells showing their potential to differentiate into adipocytes, osteoblasts, and chondrocytes (scale bar = 100 μm).

In human BM, LNGFR and THY-1 surface makers allow the isolation of highly enriched MSCs [20]. To isolate MSCs from synovium, we digested synovium with collagenase and collagenase-treated cells were stained with antibodies against LNGFR and THY-1 (Fig 1A). We identified four populations, corresponding with these markers expression (Fig 1B). To analyze the clonogenic potential of four populations, we performed the CFU-F assay for each population cells. After 2 weeks of culture, we counted the number of colonies containing more than 50 cells. Cells that were negative for both LNGFR and THY-1 (black) did not form colonies, whereas cells that were positive for both markers formed many colonies (Fig 1C). In the LNGFR^+^THY-1^-^ (green) and LNGFR^-^THY-1^+^ (blue) populations, cells formed few colonies and had a flat morphology (data not shown). Cells in the LNGFR^+^THY-1^+^ (red) population were spindle-shaped and had a robust ability to differentiate into adipocytes, chondrocytes, and osteoblasts (Fig 1D).

### SYN-MSCs have a high colony-forming ability and proliferation potential compared with BM-MSCs

**Fig 2:**
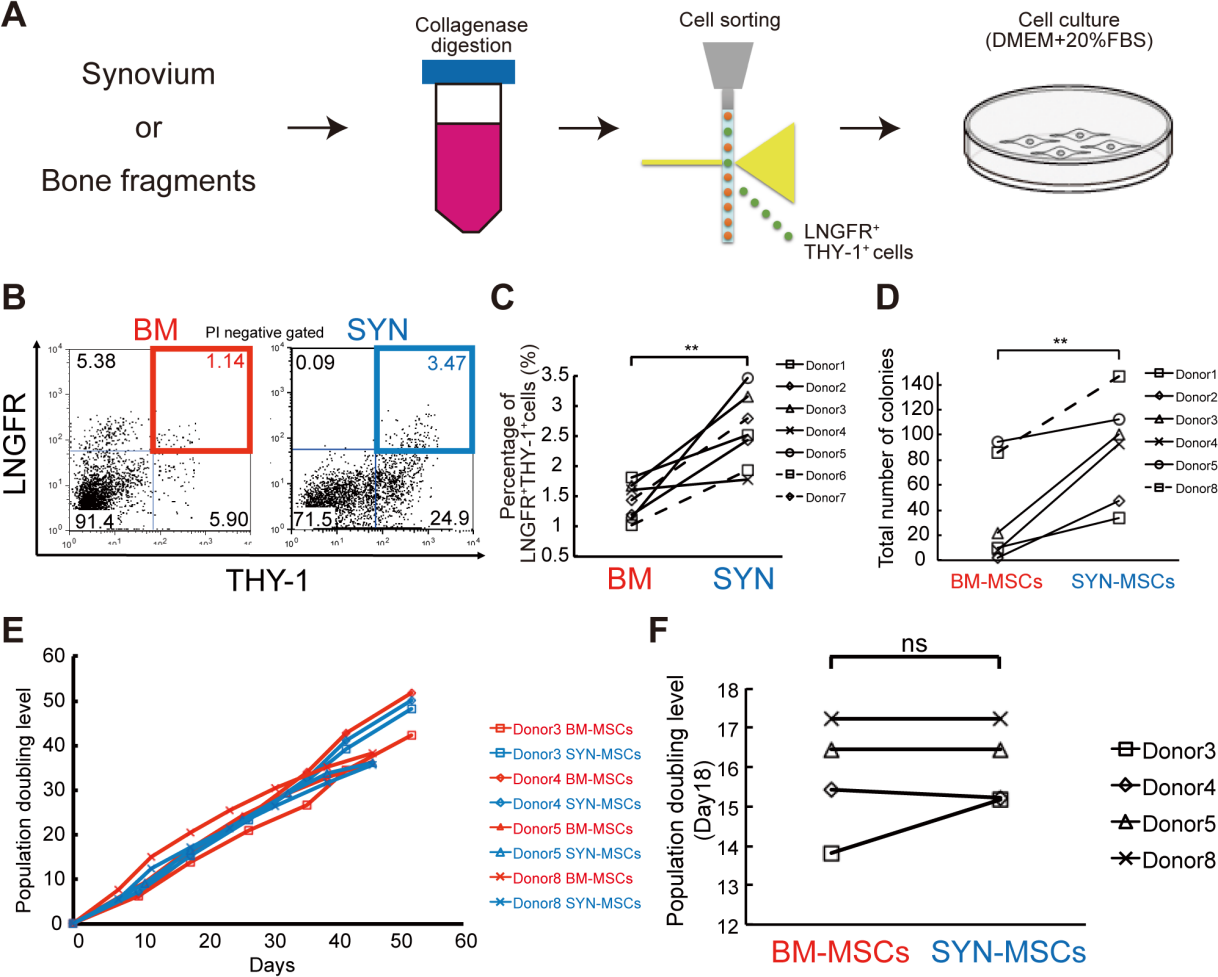
Colony-forming potential of SYN-MSCs and BM-MSCs. (A) MSCs were isolated from synovium and bone fragments. (B) Representative flow cytometric profiles of cells derived from bone marrow (BM, left) and synovium (SYN, right) of the same donor and stained for LNGFR and THY-1. PI, propidium iodide. (C) Flow cytometric analysis of the percentage of LNGFR^+^THY-1^+^ cells among BM- and SYN-derived cells (n = 7, **p < 0.01). (D) Number of colonies formed by BM-MSCs and SYN-MSCs on day 14 (n = 6, *p<0.05; **p < 0.01). (E) Representative population doubling level of BM-MSCs and SYN-MSCs. (F) Population doubling level of SYN-MSCs and BM-MSCs at day 18 (n = 4; ns, not significant).

We digested human synovum and bone fragments and stained the obtained cells with antibodies against LNGFR and THY-1, and then performed flow cytometric analysis (Fig 2A). Synovium tissue contained a significantly larger percentage of LNGFR^+^THY-1^+^ cells than BM (Fig 2B and 2C). We performed the CFU-F assay using freshly sorted cells from BM and SYN to evaluate the clonogenic potential of LNGFR^+^THY-1^+^ cells. SYN-MSCs had high clonogenic ability after 2 weeks of culture (Fig 2D). The population doubling level (Day 18) and growth kinetic analyses revealed that SYN-MSCs could be cultured for longer than 7 weeks in a similar fashion to BM-MSCs (Fig 2E and 2F).

### Expression of THY-1 correlate with cell proliferation in BM-MSCs and SYN-MSCs

**Fig 3:**
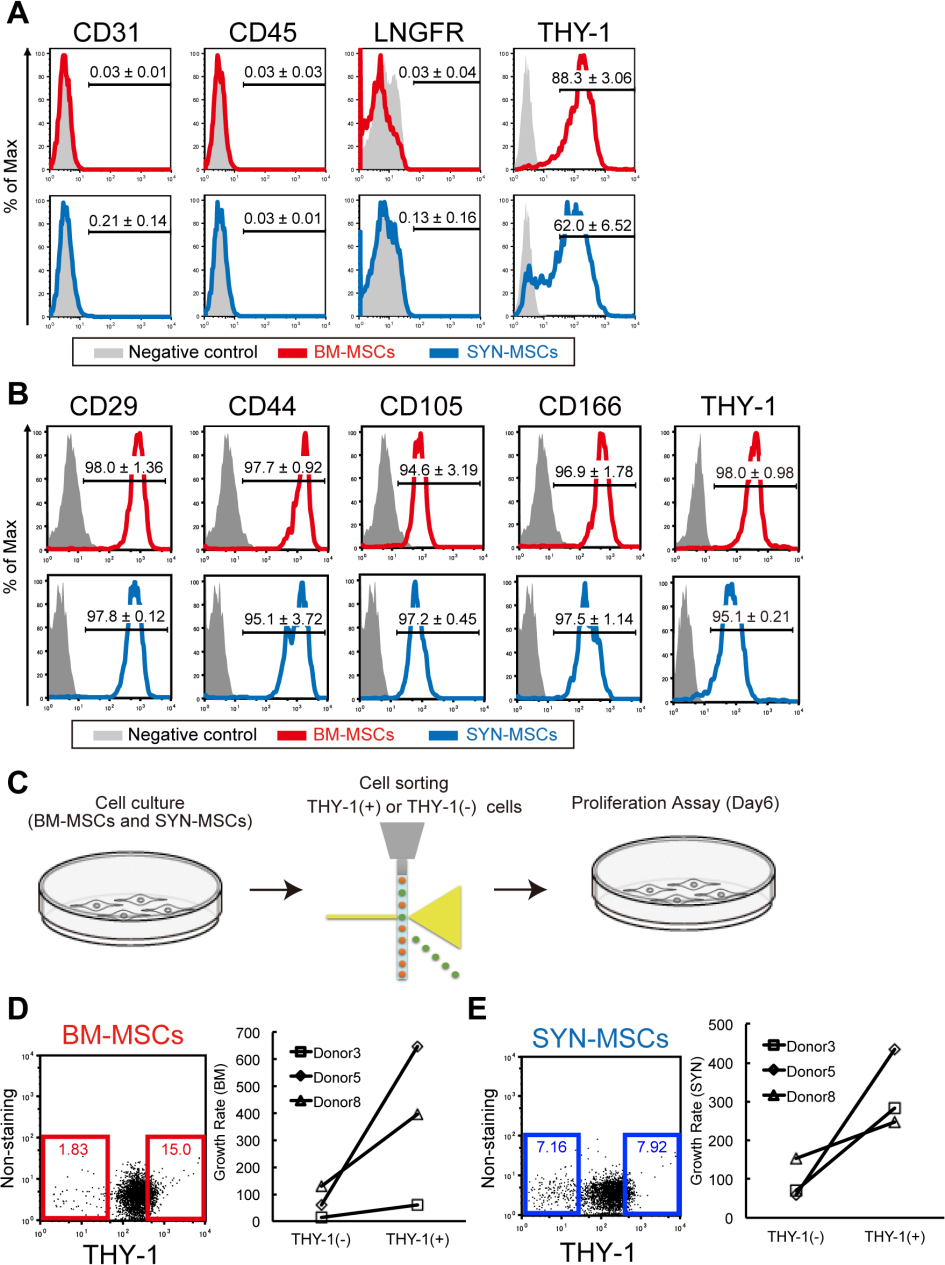
Comparison of cell surface proteins between SYN-MSCs and BM-MSCs. (A) Representative flow cytometric profiles of cells at passage 3, showing the percentage of cells expressing the antigen (red or blue line) versus the isotype control (gray). Markers: CD31, CD45, LNGFR, and THY-1. (B) The analysis of mesenchymal cell markers at passage 5, (C) Proliferation assay of cultured THY-1^+^ or THY-1^-^ cells derived from the BM-MSCs and SYN-MSCs after passage 5. (D) Flow cytometric profiles and growth rate in cultured BM-MSCs. (E) Flow cytometric profiles and growth rate in cultured SYN-MSCs.

We examined the surface epitopes of MSCs by flow cytometry. At passage 3, there were no differences in the expression of CD31 (endothelial marker), CD45 (hematopoietic marker), LNGFR and THY-1 between SYN-MSCs and BM-MSCs (Fig 3A). After passage 5, we analyze the mesenchymal markers (CD29, CD44, CD105 and CD166) (Fig 3B). The cell surface antigen of SYN-MSCs and BM-MSCs isolated from the same donor were similar profiles (n = 3). We previously reported that murine cultured BM-MSC clones with stem cell potential kept highly expressing of THY-1, whereas clones with a limited differentiation potential turned to THY-1^-^ [23]. Therefore, we isolated THY-1^+^ and THY-1^-^ cells from BM and SYN, and analyzed the cell proliferation ability of each sorted fraction (Fig 3C). We found that THY-1^+^ cells had a high proliferation ability compared with THY-1^-^ cells (Fig 3D and 3E). These data suggest that cell surface marker THY-1 was suitable for isolating MSCs in human BM but also in human SYN.

### BM-MSCs preferentially differentiate into bone, while SYN-MSCs differentiate better into adipocytes

**Fig 4:**
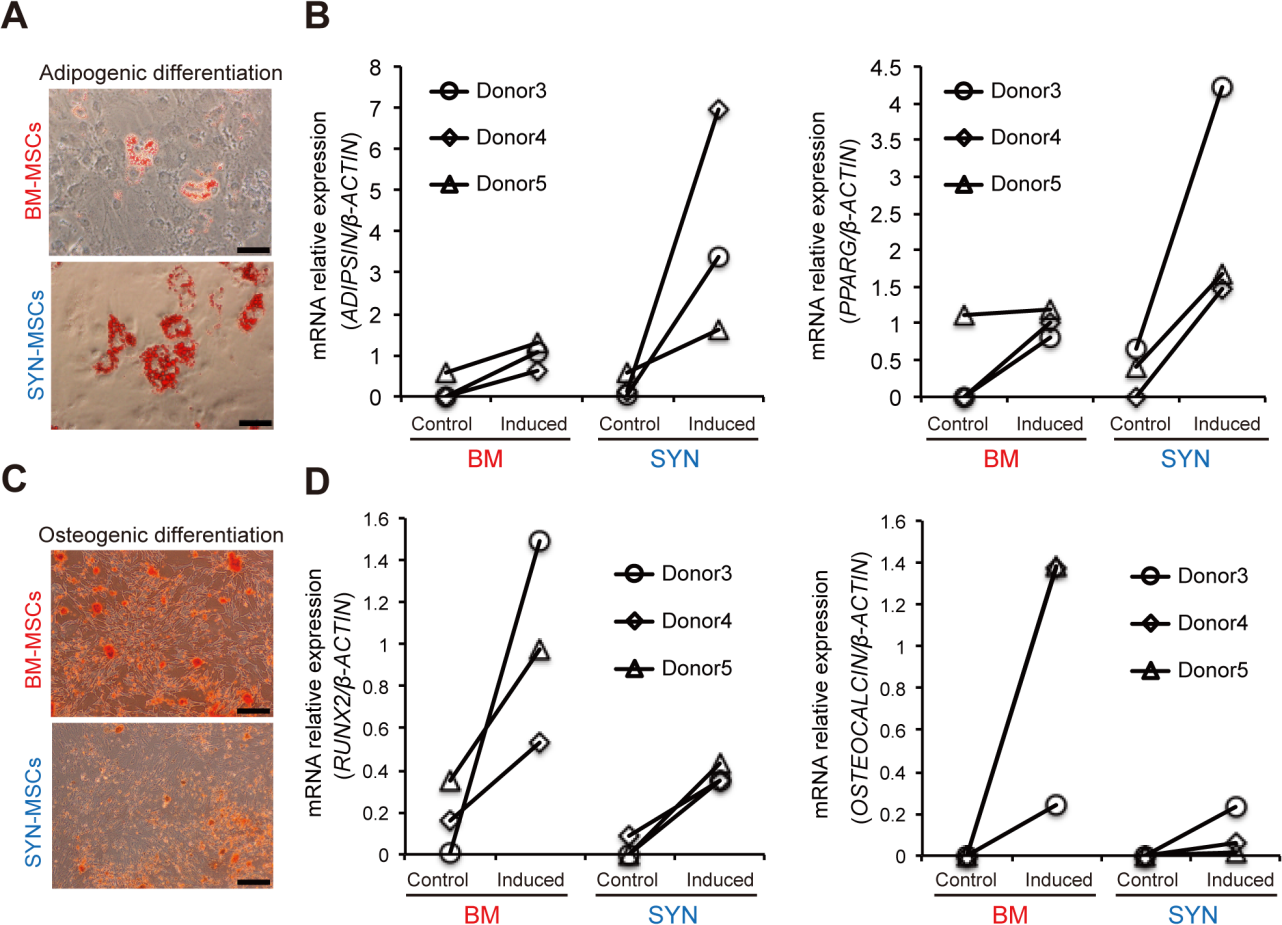
Comparison of the adipogenic and osteogenic differentiation abilities of SYN-MSCs and BM-MSCs. (A) Representative micrographs of adipocytes differentiated BM-MSCs (top) and SYN-MSCs (bottom) stained with oil red O (scale bar = 50 μm). (B) Expression ratio of *ADIPSIN and PPARG* mRNA to *β-ACTIN* mRNA following adipogenic differentiation of BM-MSCs and SYN-MSCs (n = 3). Control: non-induced MSCs, Induced: induced MSCs for adipogenic lineage. (C) Representative micrographs of osteoblasts differentiated from BM-MSCs (top) and SYN-MSCs (bottom) stained with alizarin red (scale bar = 200 μm). (D) Expression ratio of *RUNX2 and OSTEOCALCIN* mRNA to *β-ACTIN* mRNA following osteogenic differentiation of BM-MSCs and SYN-MSCs (n = 3). Control: non-induced MSCs, Induced: induced MSCs for osteogenic lineage.

We examined whether the differentiation potential of SYN-MSCs differs from that of BM-MSCs. First, we performed adipogenic differentiation of these MSCs. Although both types of MSCs effectively differentiated into adipocytes after 2 weeks, as determined by oil red O staining (Fig 4A). We analyzed the key gene expression into adipocyte lineage using TaqMan probes to examine the differentiation propensity. The expression of peroxisome proliferator-activated receptor γ (*PPARG*) and *ADIPSIN* was higher in SYN-MSCs than in BM-MSCs (more than two-fold) (Fig 4B and S1 Fig). Next, SYN-MSCs and BM-MSCs underwent osteogenic differentiation for 2 weeks (Fig 4C). The expression ratio of RUNX2 and OCN to β-actin was lower in SYN-MSCs than in BM-MSCs (less than three-fold) (Fig 4D and S1 Fig).

### Chondrogenic differentiation propensity markedly differs between SYN-MSCs and BM-MSCs

**Fig 5:**
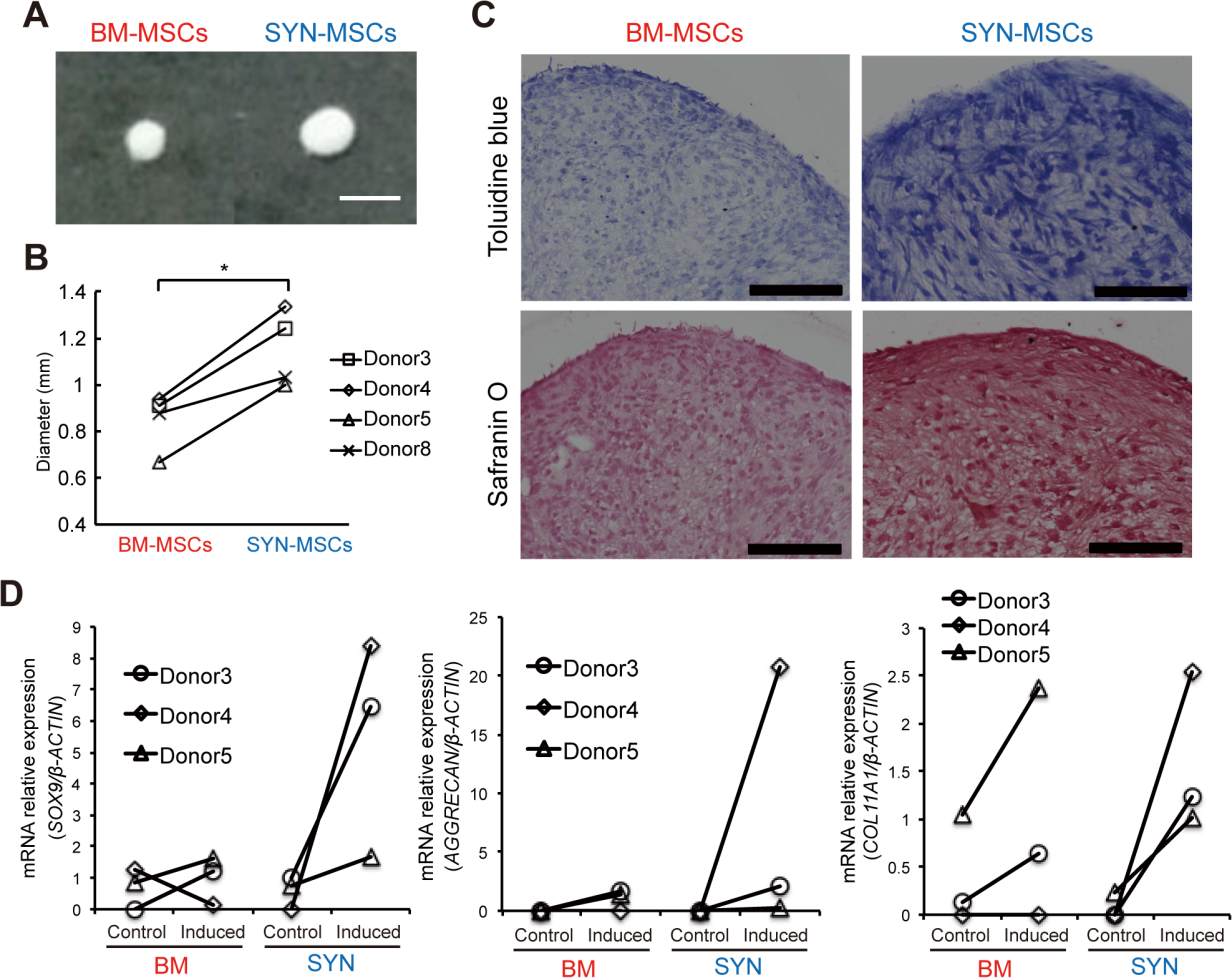
High chondrogenic differentiation ability of SYN-MSCs. (A) Representative images of chondrocyte pellets on day 21 derived from BM-MSCs and SYN-MSCs (scale bar = 1 mm). (B) Comparison of chondrocyte pellet size between BM-MSCs and SYN-MSCs (n = 4, *p < 0.05). (C) Micrographs of chondrocyte pellets derived from BM-MSCs and SYN-MSCs on day 21 stained with Toluidine blue and Safranin O (scale bar = 200 μm). (D) Expression ratio of *SOX9*, *AGGRECAN*, *and COL11A1* mRNA to *β-ACTIN* mRNA in chondrocyte pellets (n = 3). Control: non-induced MSCs, Induced: induced MSCs for chondrogenic lineage.

We performed chondrogenic differentiation of SYN-MSCs and BM-MSCs for 3 weeks. Both types of MSCs could undergo chondrogenic differentiation; however, the pellet of SYN-MSC-derived chondrocytes was 1.2-fold larger than that of BM-MSC-derived chondrocytes (Fig 5A and 5B). We also evaluated the level of chondrogenesis by staining cell pellets with Toluidine blue and Safranin O after chondrogenic differentiation. Strong staining was observed in cell pellets generated from SYN-MSCs (Fig 5C). To further verify chondrogenesis, quantitative RT-PCR was performed of the chondrogenic markers *SOX9*, *AGGRECAN (ACAN)*, and *COL11A1*. After chondrogenic induction, expression of these markers was markedly higher in SYN-MSCs than in BM-MSCs (*SOX9*: 5.5-fold, *ACAN*: 7.6-fold, *COL11A1*: 1.5-fold) (Fig 5D and S1 Fig). These results suggest that SYN-MSCs have a higher chondrogenic differentiation potential than BM-MSCs.

## Discussion

The combination of cell surface marker LNGFR and THY-1 which are originally characterized as BM-MSCs were also valuable to purify MSCs from synovium. The colony-forming ability of the LNGFR^+^THY-1^+^ population was higher than the other sub-fraction. LNGFR^+^THY-1^+^ cells exhibited a higher colony-forming potential in the CFU-F assay than that of BM-derived cells. LNGFR and THY-1 are expressed in a variety of other human tissues including placenta, adipose tissue, heart, brain, and liver [20,24–27]. Among human cells, LNGFR is predominantly expressed in melanoma-initiating cells and transit-amplifying cells [28–30]. LNGFR expression may have a common function in rapidly proliferating cells. Analyses of sorted cells revealed that the LNGFR and THY-1 markers could be used to isolate MSCs from human tissues.

We compared the surface marker expression and differentiation potential of MSCs according to the tissue from which they were derived. The osteogenic differentiation capacity of SYN-MSCs was lower than that of BM-MSCs. On the other hand, the chondrogenic differentiation capacity of SYN-MSCs was high. MSCs are shown to specify lineage and commit to phenotypes with extreme sensitivity to *in vivo* microenvironment and tissue-level elasticity [31]. The synovium supports adjacent cartilage and protects bone tissue from mechanical stresses. The characteristics of SYN-MSCs may be affected by the extracellular matrix and physical stress. THY-1 is a glycophosphatidylinositol anchored conserved cell surface protein and is used to identify MSCs. In murine MSCs, THY-1 is a key marker of multipotency [23], and besides, the expression significantly differs between human perichondrocytes and chondrocytes; its expression level is low in the latter cells [32]. The proliferation assay confirmed that THY-1^+^ cells had a high proliferation ability compared with THY-1^-^ cells in cultured SYN-MSCs and BM-MSCs. THY-1 can probably be used as a negative marker to identify MSCs which committed to chondrocytes. Taken together, SYN-MSCs may have a high chondrogenic differentiation potency because of the external environment, and immature SYN-MSCs could be isolated with THY-1 marker.

Adipose tissue-derived MSCs have a higher adipogenic differentiation potential and lower osteogenic and chondrogenic differentiation potentials than BM-MSCs [18,33]. Adipose tissue contained the MSCs that express LNGFR and THY-1 markers [20]. Synovium specimens obtained during operations contain adipose tissues; therefore, the adipogenic potential of SYN-MSCs may have been enhanced by adipose tissue-derived MSCs in this study. We used BM cells that were enzymatically isolated from bone fragments, not a BM aspirate. It is difficult to obtain MSCs from BM aspirates owing to the quality of samples. It was reported that human trabecular bone-derived cells obtained by collagenase digestion are similar to BM aspirate-derived MSCs in terms of their proliferation ability and chondrogenic, adipogenic, and osteogenic differentiation potentials [34]. These contents would not significantly change the results regarding the properties of SYN-MSCs. Therefore, this study has a relevance to the true properties of tissue-derived stem cells.

Because of many aged patients suffer from osteoarthritis (OA), regenerative therapy using MSCs has large hope in the world. When the MSCs are used in autograft, the cell isolation efficiency from tissue is an important problem. MSCs that express the same surface antigens differ in terms of their differentiation potential according to the tissue from which they are derived. Therefore, the origin of MSCs must be carefully considered before they are transplanted. Recently reports showed that the degree of inflammation was correlated with MSC isolation efficiency [35]. Because there are millions of OA patients, the synovium from OA patients are large source to isolate MSCs, similarly to cartilage injury and meniscal lesion [36]. It is an essential mission to isolate the MSC stably from SYN for facilitating regenerative medicine.

## Conclusion

The LNGFR^+^THY-1^+^ cell population of synovium forms many colonies in the CFU-F assay. LNGFR^+^THY-1^+^ SYN-MSCs have a high chondrogenic differentiation ability. SYN-MSCs may be a suitable cell source for cartilage regeneration

## Supporting Information

S1 Fig Quantitative RT-PCR assay using FAST SYBR Green reagents. Expression ratio of tergeted mRNA to *β-ACTIN* mRNA following key gene. (A) *PPARγ* for adipogenic differentiation. (B) bone sialoprotein (*BSP*) for osteogenic differentiation. (C) *AGGRECAN*, (D) *COL2A1*, and (E) *COL10A1* for chondrogenic differentiation.
(TIF)

S1 Table List of tissue sample information and actual value of isolated LNGFR^+^THY-1^+^ cells. (DOCX)

S2 Table List of TaqMan gene expression assays from Applied Biosystems. (DOCX)

S3 Table List of gene-specific primers used for quantitative RT-PCR (FAST SYBR Green). (DOCX)
