# Supplementary figures and images for: Purified Human Synovium Mesenchymal Stem Cells as a Good Resource for Cartilage Regeneration
Source: PLoS One. 2015 Jun 8;10(6):e0129096. doi: 10.1371/journal.pone.0129096 (PMC4459808; doi:10.1371/journal.pone.0129096)

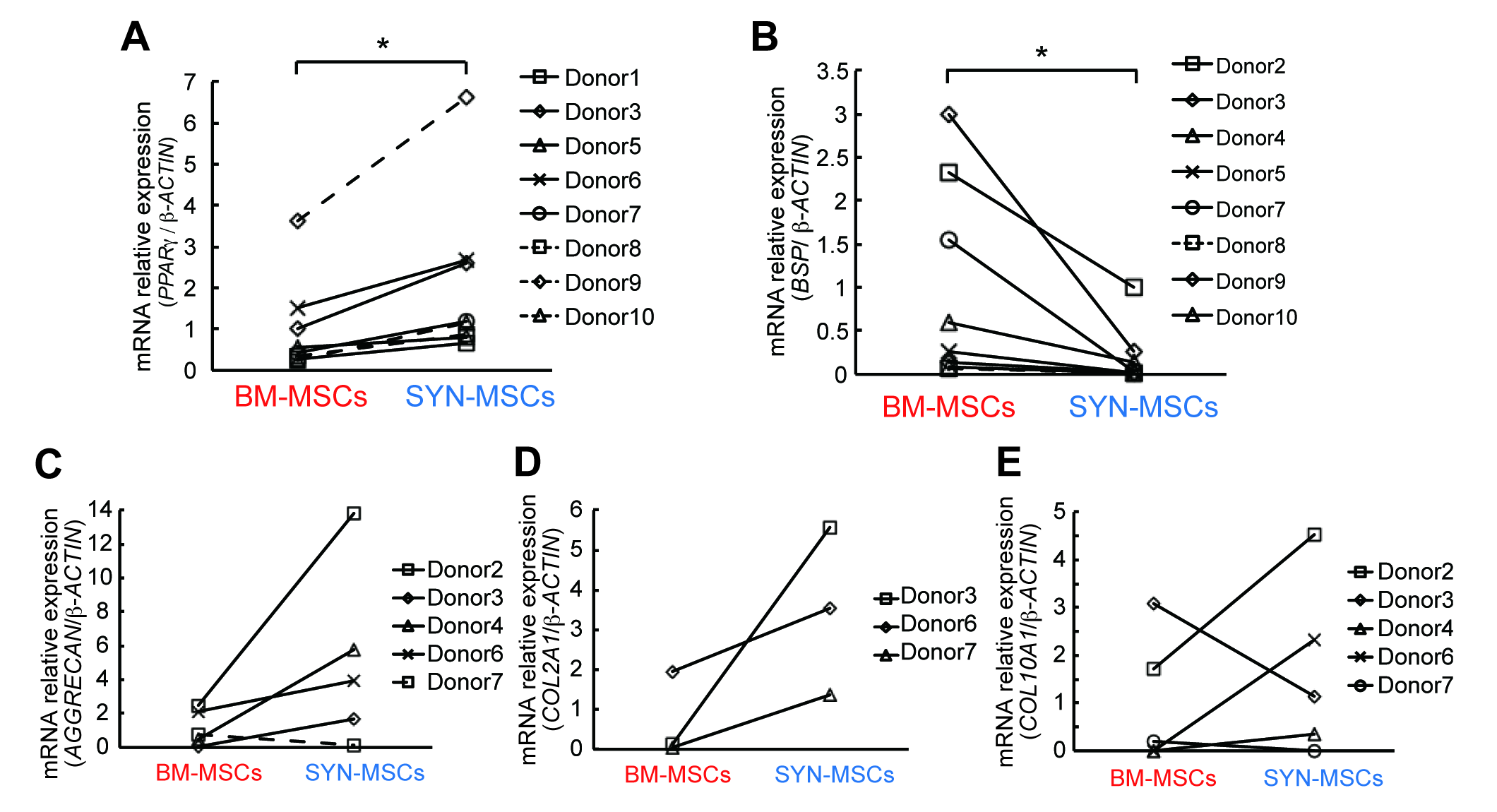

Supplement: S1 Fig — Expression ratio of tergeted mRNA to β-ACTIN mRNA following key gene. (A) PPARγ for adipogenic differentiation. (B) bone sialoprotein (BSP) for osteogenic differentiation. (C) AGGRECAN, (D) COL2A1, and (E) COL10A1 for chondrogenic differentiation. (TIF) [file pone.0129096.s001.tif]
