# Supplementary material for: Purified Human Synovium Mesenchymal Stem Cells as a Good Resource for Cartilage Regeneration
Source: PLoS One. 2015 Jun 8;10(6):e0129096. doi: 10.1371/journal.pone.0129096 (PMC4459808; doi:10.1371/journal.pone.0129096)
Supplement: S1 Table — (DOCX) [file pone.0129096.s002.docx]

**S1 Table.**

|  | Age | Gender | Operative procedure | LNGFR^+^THY-1^+^ population ratio | Sorted cell number (cells) |
| --- | --- | --- | --- | --- | --- |
| Donor 1 | 72 | Female | Osteoarthritis | BM: 1.81%, SYN: 2.52% | BM: 6000, SYN: 8000 |
| Donor 2 | 70 | Female | Osteoarthritis | BM: 1.19%, SYN: 2.43% | — |
| Donor 3 | 79 | Female | Osteoarthritis | BM: 1.68%, SYN: 3.15% | BM: 3508, SYN: 9995 |
| Donor 4 | 76 | Female | Osteoarthritis | BM: 1.60%, SYN: 1.78% | BM: 9406, SYN: 8992 |
| Donor 5 | 80 | Female | Osteoarthritis | BM: 1.14%, SYN: 3.47% | BM: 7205, SYN: 10006 |
| Donor 6 | 78 | Female | Osteoarthritis | BM: 1.02%, SYN: 1.94% | BM: — , SYN: 1700 |
| Donor 7 | 75 | Female | Osteoarthritis | BM: 1.49%, SYN: 2.80% | BM: 1035, SYN: 1200 |
| Donor 8 | 76 | Male | Osteoarthritis | BM: 1.33%, SYN: 17.3% | BM: 5129, SYN: 5502 |
| Donor 9 | 81 | Female | Osteoarthritis | BM: 1.32%, SYN: 0.65% | BM: 5002, SYN: 5012 |
| Donor 10 | 66 | Female | Osteoarthritis | BM: 1.24%, SYN: 1.09% | BM: 2069, SYN: 702 |
