# Supplementary material for: Purified Human Synovium Mesenchymal Stem Cells as a Good Resource for Cartilage Regeneration
Source: PLoS One. 2015 Jun 8;10(6):e0129096. doi: 10.1371/journal.pone.0129096 (PMC4459808; doi:10.1371/journal.pone.0129096)
Supplement: S2 Table — (DOCX) [file pone.0129096.s003.docx]

**S2 Table.**

| Gene symbol | TaqMan gene code | Application | Amplicon length (bp) | Species specificity |
| --- | --- | --- | --- | --- |
| PPARG | Hs01115513_m1 | Adipo | 90 | Human |
| ADIPSIN | Hs00157263_m1 | Adipo | 72 | Human |
| RUNX2 | Hs00231692_m1 | Osteo | 116 | Human |
| OCN | Hs01587814_g1 | Osteo | 138 | Human |
| SOX9 | Hs01001343_g1 | Chondro | 101 | Human |
| ACAN | Hs00153936_m1 | Chondro | 91 | Human |
| COL11A1 | Hs01097664_m1 | Chondro | 56 | Human |
| ACTB | Hs01060665_g1 | All samples | 63 | Human |
