# Supplementary material for: Purified Human Synovium Mesenchymal Stem Cells as a Good Resource for Cartilage Regeneration
Source: PLoS One. 2015 Jun 8;10(6):e0129096. doi: 10.1371/journal.pone.0129096 (PMC4459808; doi:10.1371/journal.pone.0129096)
Supplement: S3 Table — (DOCX) [file pone.0129096.s004.docx]

**S3 Table.**

| Primer |  | Sequence |
| --- | --- | --- |
| *β-ACTIN* | Forward | CACGGCTGCTTCCAGCTC |
|  | Reverse | CACAGGACTCCATGCCCAG |
| *PPARɤ* | Forward | GCTCTAGAATGACCATGGTTGAC |
|  | Reverse | ATAAGGTGGAGATGCAGCTC |
| *BSP* | Forward | AAACGAAGAAAGCGAAGCAGAA |
|  | Reverse | GCTGCCGTTGCCGTTTT |
| *AGGRECAN* | Forward | GAAAGGCATCGTCTTCCATT |
|  | Reverse | ACGTCCTCACACCAGGAAAC |
| *COL2A1* | Forward | TCACGTACACTGCCCTGAAG |
|  | Reverse | TGCAACGGATTGTGTTGTTT |
| *COL10A1* | Forward | AATGCCCACAGGCATAAAAG |
|  | Reverse | AGGACTTCCGTAGCCTGGTT |
